# Preoperative embolization of dual arterial supply in extralobar pulmonary sequestration: a case report and literature review

**DOI:** 10.1186/s42155-025-00640-0

**Published:** 2026-01-26

**Authors:** Sandra Gad, Michael Mohnasky, Nima Kokabi, Zachary Schrank, Austin Evans, Benjamin Haithcock, Danielle O’Hara, Patrick Brown, Andrew Caddell, Christopher Goddard, Bahareh Gholami, Ali Afrasiabi, Alex Villalobos

**Affiliations:** 1 Division of Vascular and Interventional Radiology, Department of Radiology, University of North Carolina at Chapel Hill, 101 Manning Drive, Chapel Hill, NC 27514 USA; 2 School of Medicine, University of North Carolina at Chapel Hill, Chapel Hill, USA; 3 Department of Surgery, Division of Cardiothoracic Surgery, University of North Carolina at Chapel Hill, Chapel Hill, NC USA; 4 Edward Via College of Osteopathic Medicine, Blacksburg, VA USA

**Keywords:** Pulmonary sequestration, Embolization, Extralobar, Congenital lung malformations

## Abstract

Pulmonary sequestration (PS) is the second most common pulmonary congenital malformation, which involves non-functional lung tissue that lacks communication with the tracheobronchial tree and is supplied by aberrant systemic arteries, with venous drainage to either the pulmonary or systemic venous system. This anatomic malformation increases patients’ risk of recurrent infection due to the lack of robust gas exchange. Hence, prompt intervention is warranted for favourable outcomes. Surgical resection is the gold standard treatment for PS. However, embolization of aberrant arteries prior to surgery is a promising adjunct to reduce the risk of intraoperative hemorrhage associated with the aberrant arterial supply. Here we report a case of a 47-year-old man with suspected symptomatic extralobar sequestration dual feeders from a subclavian common trunk with an anomalous pulmonary arterial connection. The patient underwent preoperative embolization of feeding and draining vessels using low-profile plug occluders. The patient tolerated embolization and surgical resection with < 50 ml blood loss. Two-month post-operative imaging demonstrated the stable position of plug occluders. This case highlights the role of preoperative embolization with low-profile plug occluders as a safe and effective strategy for achieving hemodynamic control and minimizing intraoperative bleeding risk in anatomically complex pulmonary sequestrations.

## Introduction

Pulmonary sequestration (PS) is a rare congenital anomaly, accounting for 0.15–6.40% of all congenital lung malformations [1, 2]. In PS, a non-functional portion of the lung that does not connect to the tracheobronchial system is supplied by an anomalous vascular supply [1, 2]. Most commonly, the anomalous vascular supply for PS arises systemically, such as from the thoracic aorta or the abdominal aorta [3, 4]. However, other non-systemic branches can feed PS. The classification of PS is based on its anatomic location and can be classified as intralobar or extralobar. Intralobar PS is the most common and occurs within the normal pleural envelope of the functional lung — sharing both its arterial blood supply and venous drainage with the surrounding functional lung tissue. Intralobar PS is typically located in the posterior basal segment of the left lower lobe and is often asymptomatic [1, 2]. Extralobar PS, on the other hand, has a separate visceral pleura and venous drainage [5, 6]. If significant in size, extralobar PS can often present earlier in life with respiratory distress, heart failure (due to right-to-left shunt), and concomitant congenital anomalies [6]. In terms of supply, both the intra- and extralobar PS are usually supplied from the descending aorta, or infradiaphragmatic aorta, or the coronary artery in rare cases [7]. For the intralobar type, it typically drains into the inferior pulmonary vein, whereas the extralobar type drains via the azygous vein or, in rare cases, the portal vein [7].

Due to the anomalous blood supply and its potential to cause complications such as hemoptysis and/or infection, management of PS is often pursued, and most commonly so via surgical excision [6]. Because these anomalous vessels can be numerous, tedious, and fragile, adjunct pre-operative techniques, such as endovascular embolization, can be requested by thoracic surgeons to aid with anomalous vessel location and further mitigate serious peri-operative hemorrhagic risks [6].

In this context, embolization of PS’s high-flow feeding vessels prior to surgery or, in less frequent circumstances, as a definitive therapy alternative to surgery has been reported [8–10]. Nevertheless, there remains a paucity of literature reporting on the effectiveness of pre-operative embolization. Here we report a case of a patient with a rare extralobar PS, with two prominent arterial feeding vessels, and an anomalous pulmonary arterial connection who underwent embolization using a novel application of low-profile braided plug occluder devices prior to surgical excision of the PS as part of coordinated planning between interventional radiology and thoracic surgery. Together, these features highlight the multidisciplinary and technical considerations that may be required for optimal management of anatomically complex PS.

## Case report

**Fig. 1:**
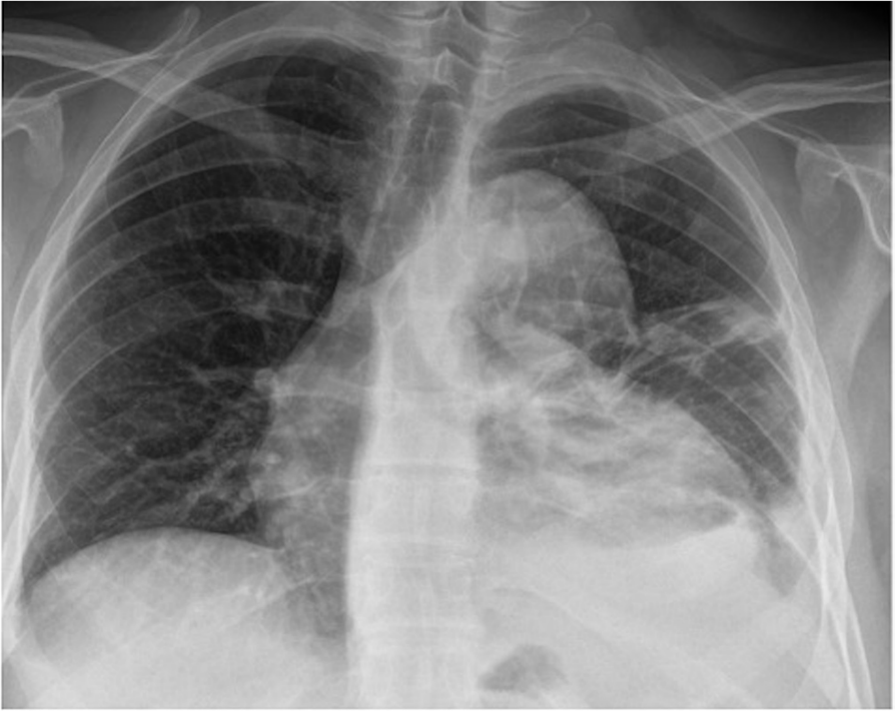
Chest radiograph demonstrating a left upper peri-mediastinal mass-like finding, with a left pleural effusion

**Fig. 2:**
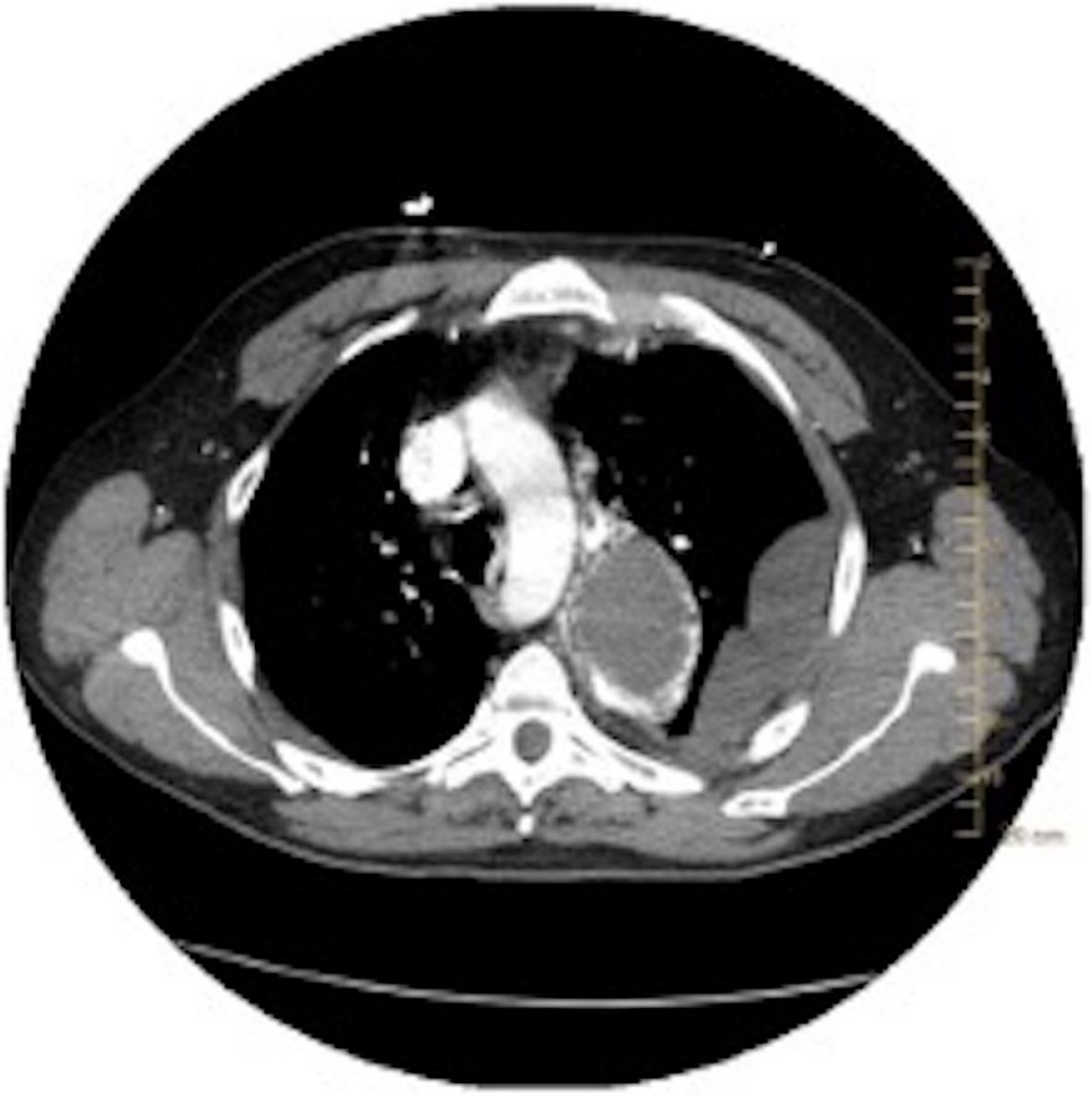
Axial image of CT chest with contrast demonstrating the posteromedial left upper lobe loculated fluid collection, concerning for an intralobular pulmonary sequestration

**Fig. 3:**
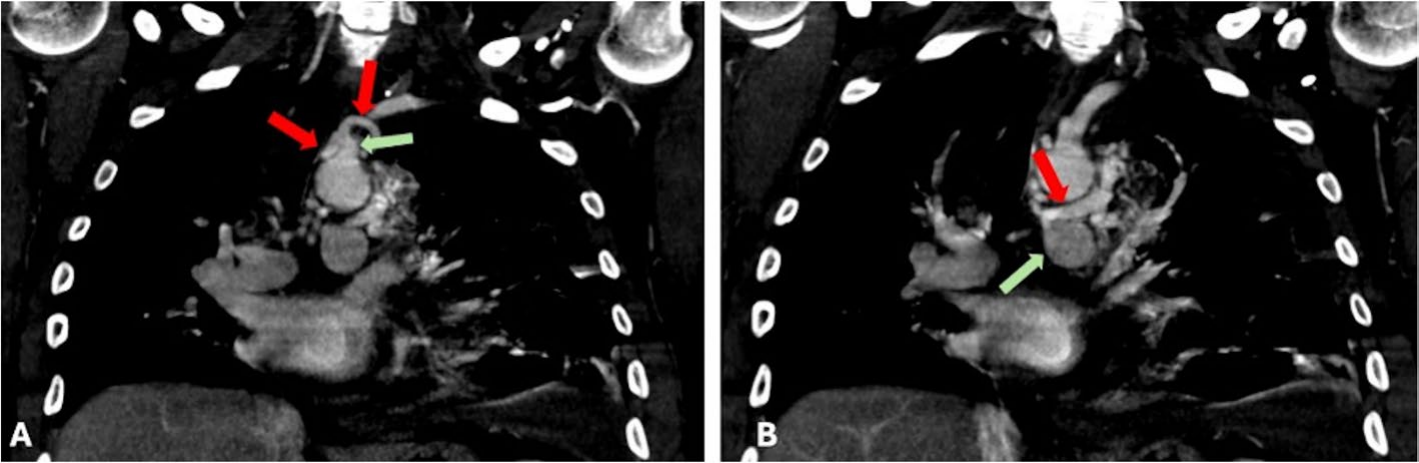
**A** Coronal image of CT chest with contrast demonstrating the two feeding arteries (red arrows) arising from the proximal left subclavian artery. Their common trunk is noted by the green arrow. **B** Coronal image of the CT chest with contrast demonstrating the draining vein (red arrow) connecting to the proximal left main pulmonary artery (green arrow)

The patient is a 47-year-old male with no known medical history. He presented to a local emergency department with 8 days of progressive left-sided chest pain, fever, and worsening cough. Chest radiograph demonstrated a left upper peri-mediastinal mass that prompted further cross-sectional imaging (Fig. 1). Subsequent Computed Tomography Angiography (CTA) demonstrated a posteromedial left upper lobe loculated fluid collection with surrounding feeding blood vessels — concerning for an infected intralobar PS with a vascular communication with the left subclavian artery and the left main pulmonary artery (Fig. 2). Additionally, there was a reactive partially loculated moderate left pleural effusion concerning for empyema (Figs. 2 and 3). Given the degree of PS inflammation, the size of the feeding vessels, and the surrounding left empyema, it was felt that there was a heightened risk of intraoperative bleeding. Therefore, interventional radiology was consulted for preoperative embolization of the PS anomalous vasculature. Preprocedural discussions with cardiothoracic surgery informed the LOBO plug placement approach. Plugs were deliberately positioned 1–2 cm away from the vessel origin to permit the surgeon the option to ligate either proximal or distal to the plug, depending on intraoperative findings. If ligation proximal to the plug was not performed, ligation occurred just distally to the plug to reinforce the expected vascular control afforded by the plug. Feedback from the surgical team indicated this approach maximized the ease and options of the surgical procedure, further emphasizing the importance of a multidisciplinary discussion prior to embolization.

## Embolization

Embolization was performed in a stepwise fashion; the systemic arterial feeders were embolized first to reduce inflow, followed by occlusion of the draining vessel [11, 12]. Under conscious sedation, left radial arterial access was obtained via standard Seldinger technique under ultrasound (US) guidance, while systemic venous access was achieved through the right common femoral vein in the same manner.

The left subclavian artery just medial to the vertebral artery was carefully catheterized using a 5 French Envoy catheter (Codman, Raynham, Massachusetts, USA). Angiography demonstrated two prominent arterial feeding vessels into the PS (one lateral while the other medial coursing) with a shared common trunk that arose from the proximal left subclavian artery. Carefully, the catheter was advanced into this common trunk, and angiography demonstrated high flow, enlarged, and tortuous arterial branches supplying the extralobar PS, with the draining vein connecting to the left main pulmonary artery.

**Fig. 4:**
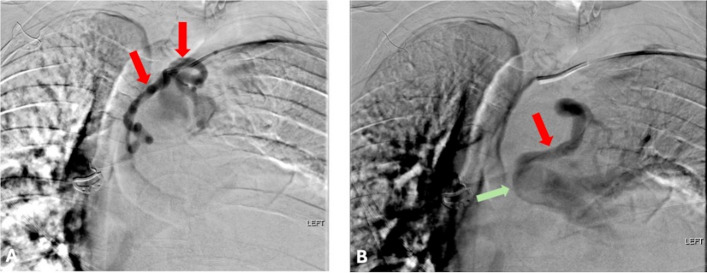
**A** Angiography demonstrating the medial and lateral high-flow feeding arteries (red arrows) arising from the subclavian artery. **B** Angiography demonstrating the feeding vein connecting to the left main pulmonary artery (green arrow)

**Fig. 5:**
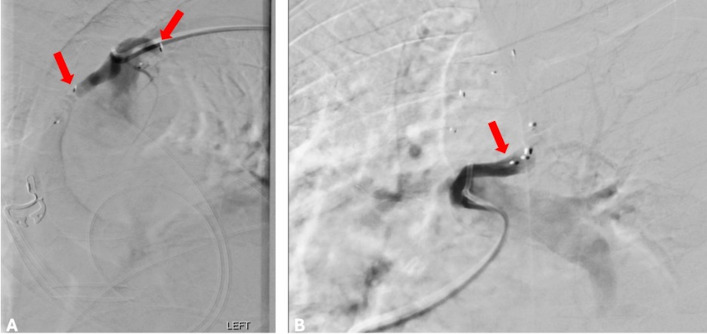
**A** The medial and lateral feeding artery branches embolized using a LOBO-3 vascular occluder device (red arrows). **B** The draining vein embolized using two LOBO-5 vascular occluder devices (red arrow)

The medial feeding artery branch measuring 3.1 mm was catheterized using a 2.9 French Sendero microcatheter (SENDERO; Okami Medical, San Diego, California) and embolized at its proximal segment using a LOBO-3 vascular occluder device (LOBO; Okami Medical, San Diego, California). Post-embolization angiography demonstrated complete cessation of flow within the medial arterial vessel and now preferential flow via the lateral feeding arterial branch. Given the acute angulation of this lateral feeding branch, the Envoy catheter was exchanged for a 5 French Vanschie catheter (Cook Medical, Bloomington, IN), and the lateral branch was selected with the 2.9 French Sendero microcatheter. Then, the proximal segment of the lateral arterial feeding branch, measuring 3.6 mm, was embolized using a LOBO-3 vascular occluder device. Subsequent post-embolization angiography demonstrated complete cessation of flow within both feeding arterial branches (Figs. 4 and 5).

The pulmonary arterial system was subsequently catheterized using 5 French Omni Flush (AngioDynamics, Latham, NY) and Envoy catheters. The patient demonstrated normal cardiac anatomy. Venous access was performed through the right common femoral vein, and the catheter was advanced sequentially through the right atrium and right ventricle into the pulmonary artery to evaluate and confirm the anomalous draining vein connection. Angiography of the left main pulmonary artery demonstrated a single large draining vein measuring 5.1 mm connecting to the proximal left main pulmonary artery. This vessel was catheterized using a 5 F Envoy catheter and embolized using LOBO-5 vascular occluder device (LOBO; Okami Medical, San Diego, California). Post-embolization angiography demonstrated a subtle, now more prominent, additional branch connecting to the PS just proximal to the recently deployed plug occluder. Therefore, an additional LOBO-5 plug was deployed just proximal to the origin of this branch. Subsequent post-embolization angiography demonstrated complete cessation of flow within the pulmonary artery branch connecting to the PS (Fig. 5).

Lastly, hemostasis was achieved at the left wrist and right groin access sites using a radial compression device and manual compression, respectively.

## Outcome

**Fig. 6:**
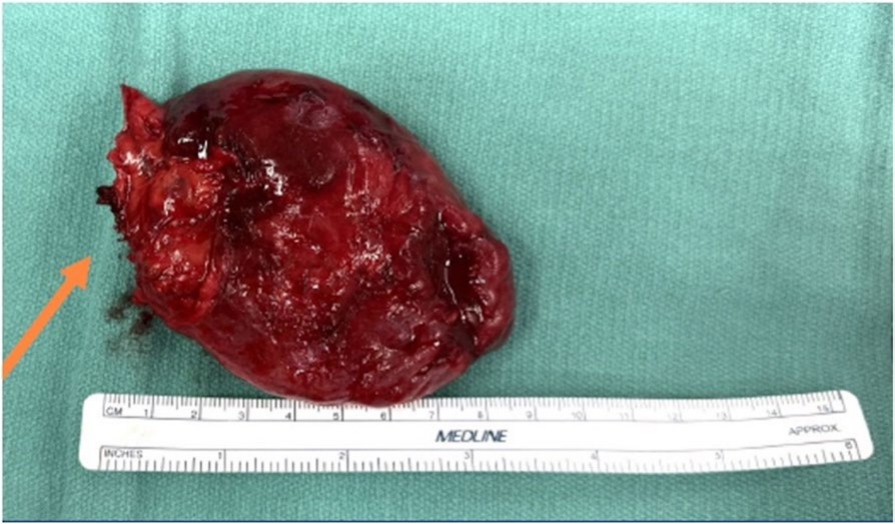
Gross photograph of the specimen demonstrates a segment of extralobar sequestration

**Fig. 7:**
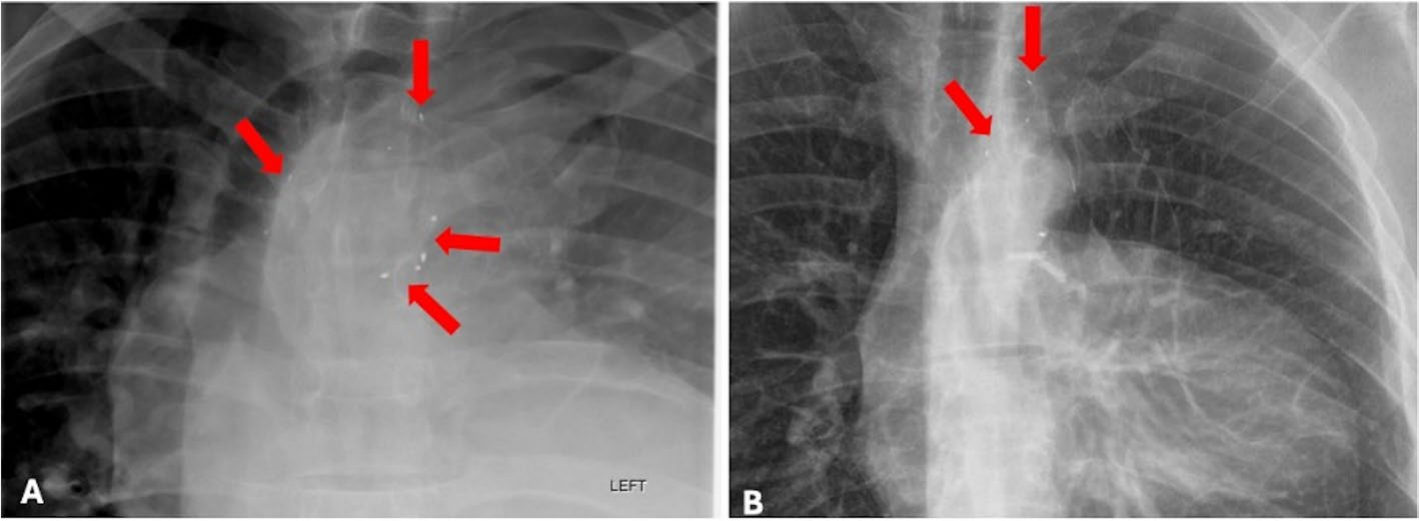
**A** Immediate post-embolization on-table radiograph demonstrating plug occluder positions (red arrows). **B** The 2-month follow-up chest radiograph demonstrating unchanged positioning of the unresected plug occluders

Immediately post embolization, the patient was transferred to the thoracic surgery team for same-day PS excision and decortication. The mass was separated from the left upper lobe without the need for parenchymal dissection, thereby representing an extralobar mass (Fig. 6). Moreover, the pericardium appeared to be absent—a rare but described association with extralobar PS [6, 13]. The draining vein plug occluders were resected with the PS. Overall, the patient tolerated both the embolization and surgical procedures well, with an estimated blood loss of 50 mL during the surgical procedure. Concurrent pneumonia and empyema were treated inpatient with vancomycin and cefepime. When pleural fluid cultures matured and revealed Gram-negative coccobacilli, he was transitioned to an antibiotic regimen of Augmentin for 14 days. He was subsequently discharged without complications on post op day 3. No embolization or surgical-related complications were noted during the routine 2-month postoperative follow-up. A routine clinic chest radiograph demonstrated the stable position of the unresected plug occluders (Fig. 7).

## Discussion

The management of PS can be complex, requiring a multidisciplinary approach to optimize patient outcomes. While surgical resection remains the definitive treatment of choice, embolization of anomalous vessels prior to surgery has emerged as a valuable adjunct therapy capable of minimizing perioperative risks. This case report presents a patient with extralobar PS fed by two high flow feeding arteries arising from the left subclavian artery, a rare vascular presentation, and a draining vein into the medial aspect of the left main pulmonary artery. This patient’s PS subsequently underwent a successful pre-operative embolization using a low-profile braided plug occluder device. This case report further highlights the benefits of preoperative embolization in patients with PS.

Based on limited case reports and series, PS is estimated to represent 0.15–8.3% of all pulmonary anomalies [4]. Patients with PS present with a wide range of symptoms, including recurrent focal pulmonary infections, persistent chest abnormalities despite treatment, chest pain, hemoptysis, and dyspnea. In rare instances and especially in infants, PS can be associated with cardiovascular malformations [6, 13]. PS can be classified as either intralobar or extralobar based on pleural encasement [1, 2, 14].

Given the wide range of clinical presentations of PS and the paucity of data, PS is often misdiagnosed and/or missed. On plain CXR, PS can mimic a wide range of pathologies that include malignancy versus lung abscess [11]. To date, the largest retrospective registry available is the 10-year Chinese National Knowledge Infrastructure database, where the authors report that approximately 30% of PS were misdiagnosed as pulmonary cysts and/or lung cancers [3]. This is unfortunate, as missed diagnoses can lead to unwarranted interventions such as lung biopsy, drain placement, and/or unnecessary bronchoscopy [12].

A high index of suspicion for PS is key for accurate diagnosis and management. Imaging, particularly CTA of the chest, plays a vital role in confirming the diagnosis by providing an accurate visualization of the feeding arteries and draining veins.

CTA is recommended for pulmonary sequestration due to its ability to provide high-resolution axial and multiplanar reconstructions, as well as 3D reformations, with the added advantage of evaluating the lung parenchyma [6]. A study by Yue et al. evaluated 43 patients with suspected PS who underwent CTA, of whom 37 were confirmed to have PS. The study demonstrated excellent diagnostic accuracy (97.7%), sensitivity (97.3%), and specificity (100%), with a positive predictive value of 100% and a negative predictive value of 85.7%. These findings reiterate CTA’s superior capability in identifying anomalous feeding arteries and venous drainage patterns [15].

The number of feeding arteries in PS varies, with a single arterial supply being the most common. However, dual or multiple feeding arteries, though rare (and as noted in this case report), have been reported [14]. Notably, in this case, CTA revealed two primary arterial supply arteries arising from a common trunk of the subclavian artery—a highly uncommon occurrence (< 1%) [3, 4]. CTA also demonstrated a draining vein into the left main pulmonary artery. Although this vessel likely represents a systemic-to-pulmonary arterial conduit rather than a true venous structure, we refer to it as a “draining vein” to maintain consistency with current sequestration terminology, recognizing that the nomenclature definition may warrant refinement in light of this rare anatomic presentation.

In this case, information provided by the CTA was crucial in ensuring comprehensive embolization, preventing residual shunting or hemorrhage, and minimizing the risk of non-target embolic complications [16]. From a physiological perspective, embolization must address the aberrant systemic arterial feeders and anomalous draining vessels. Failure to occlude both circulations can sustain high-pressure systemic flow within the sequestration and, in turn, maintain a high risk of intraoperative hemorrhage [11]. Complete embolization of both feeding and draining vessels provides hemodynamic control, reducing perioperative morbidity and enhancing procedural safety [11].

Endovascular embolization of PS has shown promise as an adjunct to surgery, particularly in reducing intraoperative bleeding. Studies including case series and small retrospective studies have demonstrated that embolization can achieve partial versus complete regression of PS in both pediatric and adult patients [8, 10, 17–21]. However, its role as a “stand-alone” treatment modality remains limited to few reported cases [22, 23].

To date, studies involving primary endovascular embolization for PS are predominantly in the pediatric population. For example, in a small case series of six pediatric patients with symptomatic PS, the primary and definite treatment modality was coil embolization. Patients were followed up for 13 months after embolization for complete involution of the lung parenchyma of the sequestration using CT scan. They reported that PS resolved in 67% (*n* = 4), while two patients required re-embolization for complete resolution [10]. In a prospective study with a mean follow-up of 44 months, 5 neonates reported complete resolution of PS in 4 and partial regressions in one patient [20]. Complete and partial regression were defined as the total disappearance and presence of residual abnormal lung tissue on CT images, respectively [20]. In a retrospective review comparing embolization (*n* = 42) versus surgical resection (*n* = 31) in young children with PS, in the embolization group using micro coils with gel foam in some, 7% experienced complete regression, 83% partial regression and 10% no regression, with 9.5% (*n* = 4) patients developing complications after embolization [24].

Nevertheless, literature regarding the efficacy of endovascular embolization as the sole treatment modality in adults with PS remains limited. Current case reports available in the literature demonstrate complete resolution of intralobular sequestration, with no indication for subsequent surgery [14, 17, 20]. When comparing surgery to endovascular treatment, no difference in outcome was reported in any of these case reports. For example, a retrospective series of 28 patients with PS compared surgical intervention (*n* = 21) with embolization and endovascular stenting (*n* = 7). In the surgical group, two patients experienced massive intraoperative haemorrhage, while the endovascular intervention group reported no hemorrhage. In this case series, three patients underwent embolization as a definitive treatment. Among these, two achieved complete regression, and one patient experienced partial regression [12]. All 28 patients experienced no recurrences [12].

While embolization has been shown to be beneficial as a pre-operative adjunct therapy, surgical resection remains the definitive treatment for PS — particularly in cases with large, sequestrated lung tissue. Surgical approaches include conventional thoracotomy and video-assisted thoracoscopic surgery (VATS), both of which allow for segmentectomy or lobectomy. Our case report demonstrates that preoperative embolization, using a low-profile braided occluder device, significantly reduced blood loss during the subsequent resection. In this case report, the patient had an estimated blood loss of only 50 mL; in contrast with cases where missed feeding arteries have historically resulted in intraoperative blood loss exceeding 1000 mL [12]. This notable difference highlights that embolization can significantly reduce blood loss in patients with frail or aneurysmal arterial feeders, which are commonly seen in PS due to high-pressure systemic flow within the sequestrated lung tissue [22, 25].

Preoperative embolization shows promising outcomes, with a wide range of embolic agents having been previously reported [16, 19]. The aforementioned literature cases often reported a single arterial feeder to the pulmonary sequestration, with embolization often being carried out with combinations of polyvinyl chloride particles, gelatin sponge particles, and/or coils [16, 19]. In the presented case, angiography revealed two prominent arterial feeding vessels and a prominent draining vein that were subsequently embolized using a low-profile braided plug occluder device (Fig. 4). The decision to use a low-profile braided plug occluder was made after angiography demonstrated high-flow hemodynamics within the target vessels and the need for immediate, durable occlusion prior to same-day surgical resection. The unique minimal streak artifact and the segmented flexibility properties of this low-profile braided plug occluder device also allowed for easy deployment within the tortuous vasculature and the targeted landing zones, enabling the patient to undergo further imaging in the future with minimal disruption due to the device’s minimal streak artifact [26].

Vessel measurements obtained during angiography (3.1 mm and 3.6 mm for the systemic feeders, and 5.1 mm for the draining vessel) guided the selection of plugs. LOBO-3 and LOBO-5 occluders were selected, respectively, providing appropriate vessel apposition given the gradual distal tapering. LOBO plugs do not require oversizing due to their braided nitinol design, which allows circumferential wall apposition and stable anchoring [26]. This characteristic eliminates the risk of distal migration while ensuring immediate occlusion.

Lastly, while embolization-related complications, such as non-target embolization or post-embolization syndrome (characterized by fever and mild chest discomfort), are rare, they should always be considered. In this case, the patient tolerated the procedure well without procedure-related complications or adverse events. Furthermore, 2-month post-operative radiograph demonstrated that the unresected plug occluder devices remain unchanged in position, even after nearby surgical manipulation.

In conclusion, our case report underscores the importance of the multidisciplinary approach in managing PS. While the role of embolization as a “stand-alone” treatment remains debatable, its role prior to surgical intervention is promising. Long-term data regarding the efficacy of neoadjuvant embolization treatment is needed.

## Data Availability

The datasets used and/or analyzed during the current study are available from the corresponding author on reasonable request.

## Abbreviations

PS: Pulmonary sequestration
VATS: Video-assisted Thoracoscopic Surgery
US: Ultrasound
CTA: Computed tomography angiogram
